# Reconstitution of DNA repair synthesis *in vitro* and the role of polymerase and helicase activities

**DOI:** 10.1016/j.dnarep.2011.03.003

**Published:** 2011-06-10

**Authors:** Marek Sebesta, Peter Burkovics, Lajos Haracska, Lumir Krejci

**Affiliations:** a Department of Biology, Masaryk University, Kamenice 5/A7, 625 00 Brno, Czech Republic; b National Centre for Biomolecular Research, Masaryk University, Kamenice 5/A4, 625 00 Brno, Czech Republic; c Institute of Genetics, Biological Research Center, Hungarian Academy of Sciences, Temesvari krt. 62, 6726 Szeged, Hungary; d International Clinical Research Center, St. Anne's University Hospital Brno, Pekarska 53, 656 91 Brno, Czech Republic

**Keywords:** DNA repair, Recombination, DNA synthesis, Replication, Mph1, Srs2

## Abstract

The error-free repair of double-strand DNA breaks by homologous recombination (HR) ensures genomic stability using undamaged homologous sequence to copy genetic information. While some of the aspects of the initial steps of HR are understood, the molecular mechanisms underlying events downstream of the D-loop formation remain unclear. Therefore, we have reconstituted D-loop-based *in vitro* recombination-associated DNA repair synthesis assay and tested the efficacy of polymerases Pol δ and Pol η to extend invaded primer, and the ability of three helicases (Mph1, Srs2 and Sgs1) to displace this extended primer. Both Pol δ and Pol η extended up to 50% of the D-loop substrate, but differed in product length and dependency on proliferating cell nuclear antigen (PCNA). Mph1, but not Srs2 or Sgs1, displaced the extended primer very efficiently, supporting putative role of Mph1 in promoting the synthesis-dependent strand-annealing pathway. The experimental system described here can be employed to increase our understanding of HR events following D-loop formation, as well as the regulatory mechanisms involved.

## 1 Introduction

Damage to DNA in cells continuously arises either endogenously (*e.g.* from exposure to metabolically generated oxidants or replication of damaged template) or exogenously (*e.g.* from exposure to ionizing radiation or xenochemicals). Among these DNA lesions, the double-stranded breaks (DSBs) represent the most toxic form, which once left unrepaired can lead to cell cycle arrest and cell death [1].

There are two major pathways for the repair of DSBs: a non-homologous end joining (NHEJ) pathway, and a homologous recombination (HR) pathway [2]. NHEJ occurs predominantly in higher eukaryotes, or in cases where a cell lacks a homologous sequence. In this pathway the broken arms of DNA are simply rejoined, with or without processing or micro-homology-mediation, often accompanied by deletions or insertions [3]. In contrast, in HR a homologous sequence is used as a donor from which the damaged or lost sequence of the broken molecule is copied in an error-free manner.

In the early steps of HR, DSBs are resected to generate a 3′ single-stranded DNA (ssDNA) tail. This 5′ resection at the site of the break associates with the Rad50-Mre11-Xrs2 (RMX) nuclease complex, together with Sae2, Exo1 and Dna2/Sgs1 proteins [4,5]. The ssDNA tail is protected by replication protein A (RPA), and is transformed into a Rad51 nucleoprotein filament (also known as a pre-synaptic filament) with the help of recombination mediators (Rad52, Rad59 and Rad55/Rad57) [6–8], which is then capable of searching for homologous sequences [9]. However, in the case of “unscheduled” recombination, Srs2 helicase can counteract filament formation by disassociating Rad51 from ssDNA and directly competing with Rad52 [10–13]. Thus, Srs2 and Rad52 serve as HR quality controllers, ensuring normal course of recombination [14,15].

As HR proceeds, the Rad51 pre-synaptic filament invades donor duplex DNA to form a stable intermediate known as the D-loop [16,17]. This process is promoted by Rad54, a molecular motor protein that not only stabilizes the nucleoprotein filament, but also allows the search for homologous sequences in normal and chromatinized templates [18–20]. The invading strand in the D-loop structure is then extended using several components of the replication machinery, namely: DNA polymerase δ or ɛ; the proliferating cell nuclear antigen (PCNA); and its loader, replication factor C (RFC) [21–23].

HR can then proceed by either of two sub-pathways. The first of these is double-strand break repair (DSBR), in which the second end of the broken DNA is captured and stabilized by D-loop, then a second round of DNA synthesis occurs, followed by the formation of a double Holliday junction. This structure can then be resolved or dissolved, generating crossover or non-crossover products. In the alternative sub-pathway, synthesis-dependent strand-annealing (SDSA), the newly extended strand is displaced from the D-loop and annealed with its original complementary strand to generate a non-crossover product. *In vitro* and *in vivo* data suggest that the actions of helicases Mph1, Srs2, and Sgs1 are required for the displacement of the extended primer [24–27].

In contrast to the initial phases of HR, the steps occurring after D-loop formation are poorly understood. Hence, our aim in the present study was to reconstitute recombination-associated DNA repair synthesis machinery *in vitro*, capable of mimicking the *in vivo* events downstream of D-loop formation, in order to study the primer extension step during the repair of DSBs and the role of specific helicases in promoting the SDSA pathway of HR. The results both provide new information on the events downstream of D-loop formation during DSB repair, and demonstrate that the reconstituted system provides a means to explore these events (and the regulatory mechanisms involved) in detail.

## 2 Materials and methods

### 2.1 DNA substrates

Oligonucleotides were purchased from VBC Biotech with sequences shown in Table 1. Substrates were prepared as described by [28].

### 2.2 PCNA purification

PCNA was expressed in *E. coli* and purified essentially using the procedure described by Ayyagari et al. [29]. Briefly, 6 g of *E. coli* cell paste was sonicated in 30 ml lysis buffer C, consisting of 50 mM Tris–HCl (pH 7.5), 10% sucrose (w/v), the protease inhibitors EDTA (10 mM), dithiothreitol (1 mM), nonidet (0.01%, v/v), and KCl (750 mM). The crude lysate was clarified by centrifugation (100,000 × *g* for 90 min). A fraction of the proteins within the supernatant was then harvested by adding 0.21 g solid ammonium sulfate per ml, stirring for 1 h, centrifuging at 15,000 × *g* for 20 min, adding another 0.32 g/ml solid ammonium sulfate to the supernatant, stirring for 1 h, then centrifuging at 15,000 × *g* for 1 h. The resulting pellet was dissolved in 35 ml of buffer K – 20 mM K_2_HPO_4_, 20% (v/v) glycerol, 0.5 mM EDTA (pH 7.5), 0.01% (v/v) NP40, and 1 mM β-mercaptoethanol – then the mixture was applied to a 7-ml SP sepharose column (GE Healthcare Life Sciences). The flow-through was immediately loaded onto a 7 ml Q sepharose column (GE Healthcare Life Sciences), and eluted with a 70 ml linear gradient of 50–900 mM KCl in buffer K. The peak fractions were pooled and loaded onto a 1 ml hydroxyapatite column (BioRad), and proteins were eluted with a 15 ml gradient from 50 to 1000 mM KH_2_PO_4_ in buffer K. Peak fractions were again pooled, and loaded onto a 1 ml Mono Q column (GE Healthcare Life Sciences), and eluted with a 15 ml gradient of 50–900 mM KCl in buffer K. Fractions containing nearly homogenous PCNA were concentrated in a Vivaspin concentrator and stored in 5 μl aliquots at −80 °C.

### 2.3 Srs2 and Srs2^1–860^ purification

*E. coli* Rosseta cells were transformed with plasmids harboring sequences encoding Srs2 and Srs2^1–860^ fused with a His_9_ tag at the N-terminus, then the proteins were expressed and purified essentially as described by [12]. Nearly homogeneous Srs2 polypeptides were flash-frozen in liquid nitrogen in 2 μl aliquots and stored at −80 °C.

### 2.4 Polymerase δ purification

The polymerase δ complex was expressed and purified according to the method of [30] with modifications. Briefly, 100 g of cell paste was lysed in a cryo-mill, the resulting powder was re-suspended in 200 ml lysis buffer C containing 175 mM (NH_4_)_2_SO_4_ and centrifuged (100,000 × *g* for 60 min at 4 °C). The volume of cleared supernatant was measured and 40 μl of 10% (v/v) Polymin P was added per ml of lysate. After 5 min the suspension was centrifuged at 15,000 × *g* for 45 min and solid (NH_4_)_2_SO_4_ was added to the supernatant to a final concentration 0.28 g/ml. Precipitated proteins were collected by centrifugation (15,000 × *g* for 20 min at 4 °C), and the pellet was re-suspended in buffer K to yield conductivity equivalent to that of buffer K containing 25 mM KCl. The suspension was loaded onto a 25 ml SP-sepharose column, and proteins were eluted with 100 ml of buffer K containing 750 mM KCl. Protein fractions were pooled and dialyzed against buffer K containing 25 mM KCl and loaded onto a 1 ml Mono Q column. Proteins were then eluted with a 15 ml gradient of 25–500 mM KCl in buffer K. Peak fractions were pooled, diluted 3-fold, loaded onto a 1 ml Mono S column and proteins were eluted with a 15 ml gradient of 50–500 mM KCl in buffer K. The eluted proteins were pooled, concentrated to 300 μl and loaded onto a 25-ml Superdex 400 column (GE Healthcare Life Sciences), which was eluted with 25 ml buffer K containing 300 mM KCl. Nearly homogeneous polymerase δ was concentrated, flash-frozen in 2 μl aliquots, and stored at −80 °C.

### 2.5 Purification of other proteins

Rad51, Rad54 and RPA were purified according to the procedure of [31], while RFC complex, Mph1 and Pol η were purified according to [32–34], respectively.

### 2.6 D-loop and primer extension assays

Essentially, the D-loop assay was performed as described by [10]. Briefly, fluorescently labeled, radioactively labeled or unlabeled 90-mers (3 μM nucleotides) were incubated for 5 min at 37 °C with Rad51 (1 μM) in 10 μl of buffer R (35 mM Tris–HCl pH 7.4, 2 mM ATP, 2.5 mM MgCl_2_, 50 mM KCl, 1 mM DTT and an ATP-regenerating system consisting of 20 mM creatine phosphate and 20 μg/ml creatine kinase) then 1 μl of Rad54 (150 nM) was added and the mixture was incubated for a further 3 min at 23 °C. The reaction was initiated by adding pBluescript replicative form I (50 μM base pairs) in 1.5 μl, and the mixture was incubated for 5 min at 23 °C.

Next, RPA (660 nM), PCNA (6.66 nM), RFC (10 nM) and either Pol δ or Pol η (15 nM) in buffer O (20 mM Tris–HCl pH 7.5, 5 mM DTT, 0.1 mM EDTA, 150 mM KCl, 40 μg/ml BSA, 8 mM MgCl_2_, 5% (v/v) glycerol, 0.5 mM ATP and 75 μM each of dGTP and dCTP) were added, and the mixture was incubated for 5 min at 30 °C. DNA synthesis was initiated by adding start buffer (75 μM dTTP and either unlabeled dATP at 75 μM or 0.375 μCi [α-^32^P]dATP in buffer O) to a 30 μl final reaction volume. Equal amounts of all dNTPs was not necessary to use to monitor the reaction as lower amounts of [α-^32^P]dATP did not have any effect on the extension reaction. After 10 min at 30 °C, reactions were stopped with SDS (0.5% final) and proteinase K (0.5 mg/ml) at 37 °C for 3 min, and loaded onto an agarose gel (0.8%, w/v). After electrophoresis the gel was dried on DE81 paper and either exposed to a phosphorimager screen, or directly scanned for fluorescent DNA with a Fuji FLA 9000 imager, followed by analysis with Multi Gauge software (Fuji).

### 2.7 2D gel electrophoresis

D-loop formation and primer extension reactions were performed as described above, the resulting mixtures were split into two parts and electrophoretically separated in 0.8% (w/v) agarose gel in 1× TAE buffer, then lanes were excised from the gel. The lane displaying D-loops and products from one aliquot of each reaction mixture was dried, and the rest of the gel was soaked for 60 min in denaturing buffer (50 mM NaOH, 1 mM EDTA). The excised lane was loaded onto a denaturing agarose gel (1% (w/v) in 50 mM NaOH and 1 mM EDTA) and run for 6 h. The dried gel was exposed to a phosphorimager screen and visualized using a Fuji FLA 9000 imager with Multi Gauge software (Fuji).

### 2.8 Dissociation of extended primer by helicases

Primer extension reactions using Pol δ were performed as described above. When the reactions were complete, serial concentrations (1, 7, 33 and 167 nM) of Srs2, Srs2^1–860^ or Mph1 were added and the mixtures were incubated for a further 5 min at 30 °C. The reactions were stopped with SDS (0.5% final) and proteinase K (0.5 mg/ml) and loaded onto a 0.8% (w/v) agarose gel. After electrophoresis the gel was dried on DE81 paper, exposed to a phosphorimager screen, and analyzed using a Fuji FLA 9000 imager with Multi Gauge software (Fuji).

## 3 Results

### 3.1 Proteins required for extending the D-loop substrate

**Fig. 1:**
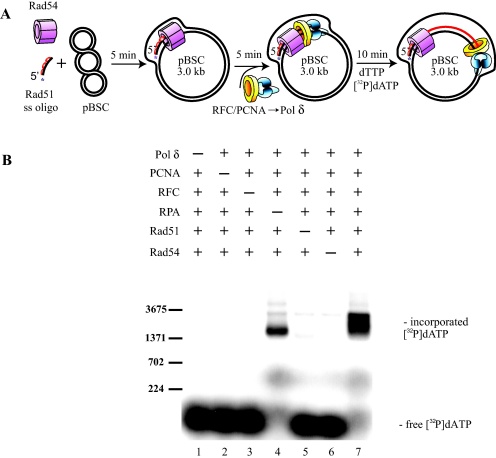
Reconstitution of recombination-associated DNA synthesis. (A) Schematic diagram of the reaction. Rad51 protein (1 μM) was mixed with ssDNA oligonucleotide D1 (3 μM nucleotides) and incubated at 37 °C for 5 min in the presence of ATP (2.5 mM). Rad54 (150 nM), depicted as oligomer [57,58] was added to the mixture and incubated for a further 3 min at RT. D-loop formation was initiated by adding supercoiled pBluescript dsDNA (50 μM as nucleotides). Next, PCNA (6.66 nM) was loaded onto the primer by RFC complex (10 nM) during 5 min incubation at 30 °C in the presence of Pol δ (15 nM), dGTP and dCTP (75 μM each). Primer extension was initiated by adding dTTP (75 μM) and [α-^32^P] dATP, followed by incubation for 10 min at 30 °C. Reactions were stopped, then the mixtures were deproteinized and analyzed. (B) Proteins required for recombination-associated DNA synthesis. The reaction was performed as described in (A) except that indicated proteins were omitted from the reaction mixtures. Labeled lambda digested with *Bst*EI was used as marker (only a subset of bands is depicted in the figure).

The D-loop represents one of the first intermediates of HR, therefore we used it as a substrate to confirm the functionality and explore the roles of components of our reconstituted DNA repair synthesis machinery. The initial part of the *in vitro* process consisted of a standard D-loop assay involving formation of the Rad51 nucleoprotein filament assembled on 90-mer oligo D1, followed by invasion of homologous super-coiled plasmid dsDNA aided by Rad54 (Fig. 1A). After the D-loop formation several factors expected to play a role in the subsequent DNA synthesis reactions were added (Pol δ, RFC and PCNA at 15 nM, 10 nM and 6.6 nM, respectively). Strong incorporation of radiolabeled dATP was only observed when all of these components were added (Fig. 1B, lane 7). However, some unspecific extension was observed in the absence of a Rad51/Rad54-mediated D-loop (Fig. 1, lanes 5 and 6). Addition of the single-strand binding protein, RPA (666 nM), enhanced the extension of the D-loop substrate, indicating that secondary structures might abrogate the synthesis of long extension products (Fig. 1, lane 4). Thus, Pol δ, RFC, PCNA and RPA are essential for primer extension (Fig. 1, lane 7), and all D-loop mediating factors (including Rad51 and Rad54) are also required for maximal and specific extension.

Next, we titrated individual components to determine the optimal reaction conditions (Supplementary Fig. 1). Addition of various concentrations of PCNA (up to 6.6 nM), at sub-equimolar concentrations (compared to Pol δ), resulted in more efficient extension (Supplementary Fig. 1, lanes 5–8). Further increase of PCNA concentration did not significantly affect the extent of DNA synthesis, but resulted in the accumulation of shorter extension products (lanes 6 and 7). Conversely, increasing RPA concentrations from 0.66 through 6.6 and 66 to 666 nM (lanes 9–12) improved the efficiency of D-loop extension. Addition of Pol δ at concentrations of 0.15, 1.5 and 15 nM resulted not only in strong stimulation of primer extension activity, but also generated fully extended products (Supplementary Fig. 1A, lanes 13–15). In contrast, the addition of 150 nM Pol δ resulted in a dramatic reduction in the efficiency of DNA synthesis (Supplementary Fig. 1, lane 16). To test the specificity of our extension assay we also used a bacterial polymerase (Klenow fragment). As shown in Supplementary Fig. 1B, Klenow fragment (500 nM) was only able to extend the D-loop substrate at concentrations 30-times higher compare to Pol δ indicating the specificity of the reaction.

To further analyze the role of RPA in the process, we performed an order of addition experiment (Supplementary Fig. 2), in which RPA was added at various points and the reaction was monitored using [^32^P]-dATP. Predictably, adding RPA before the formation of the nucleoprotein filament inhibited the process (lane 2). When RPA was added after Rad51 nucleation of ssDNA, it had no effect (lanes 3–6), irrespective of whether it was added together with Rad54, dsDNA, or either before or after loading PCNA (Supplementary Fig. 1, lanes 3–6).

### 3.2 Pol δ–PCNA interaction is essential for repair synthesis

**Fig. 2:**
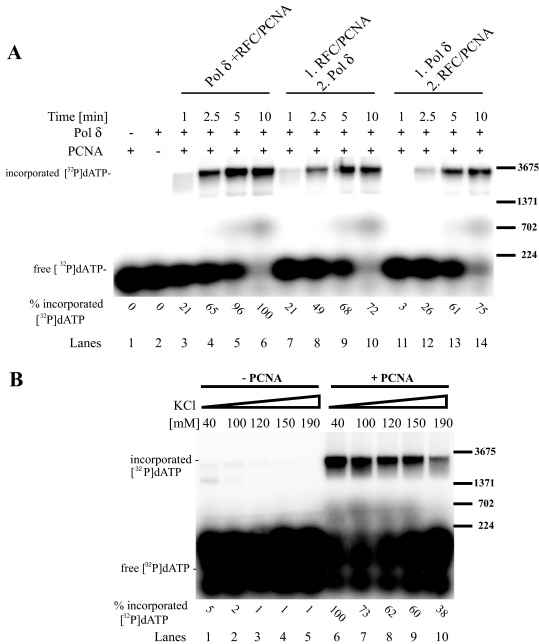
Loaded PCNA is crucial for DNA repair synthesis. (A) PCNA needs to be loaded to stimulate DNA repair synthesis. An order of addition experiment was performed in which Pol δ (15 nM) was added at the same time as (lanes 3–6), after (lanes 7–10), or before (lanes 11–14) PCNA (6.66 nM)/RFC (10 nM). Samples were withdrawn after 1, 2.5, 5 and 10 min. In control experiments indicated factors were omitted (lanes 1 and 2). (B) PCNA was required at every salt concentration. Gel showing results of D-loop assays using oligonucleotide D1 and [^32^P]-dATP in the presence of 40, 100, 120, 150 and 190 mM KCl in both the absence (lanes 1–5) and presence of PCNA (lanes 6–10). Labeled lambda digested with *Bst*EI was used as marker (only a subset of bands is depicted in the figure).

To corroborate the finding that PCNA must be loaded on DNA for primer extension to occur, we performed another order of addition experiment, again using [^32^P]-dATP to follow the reaction, taking samples 0, 1, 2.5, 5 and 10 min after its addition (Fig. 2A). When PCNA was added together with Pol δ (lanes 3–6), 65% of the [^32^P]-dATP was incorporated in reaction products within 2.5 min, but only 49% was incorporated within this time when PCNA was loaded before addition of Pol δ (lanes 7–10). A further delay was observed when Pol δ was incubated first with the D-loop, followed by addition of PCNA/RFC complex (lanes 11–14); these conditions led to 26% incorporation within 2.5 min (Fig. 2A). The effects of the salt concentration on PCNA requirements were also studied by varying the concentration of KCl in reaction mixtures (Fig. 2B) both lacking PCNA (lanes 1–5) and containing PCNA (lanes 6–10). In the absence of PCNA at 40 mM KCl, 95% less [^32^P]-dATP was incorporated than in the presence of PCNA (lanes 1 and 6). Furthermore, in the presence of PCNA, incorporation of the [^32^P]-dATP decreased with increasing salt concentrations, and only 38% incorporation was detected in mixtures with 190 mM KCl, suggesting that high salt concentrations adversely affect the Pol δ/PCNA interaction. Almost identical results were obtained from order of addition and salt dependency experiments using the φX DNA-based extension assay (Supplementary Fig. 3A and B). Overall, these findings suggest that for efficient extension PCNA must be actively loaded onto the substrate together with Pol δ and that this efficiency is salt dependent. At low salt concentrations some PCNA-independent extension was also observed.

### 3.3 The efficiency and length of D-loop extension

**Fig. 3:**
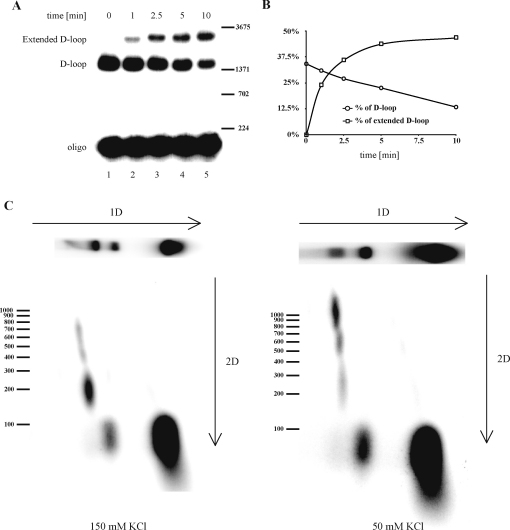
Characterization of DNA repair synthesis. (A) Extent of D-loop synthesis monitored in a time course experiment. Gel showing electrophoretically separated D-loops and products in samples withdrawn at 0, 1, 2.5, 5 and 10 min after PCNA addition. Labeled oligonucleotide was used to monitor the rate of the extension. (B) Plots showing the accumulation of extended products (percentage of extended products versus D-loops, squares) and D-loops (percentage of D-loops versus oligo, circles) presented in (A). (C) The extension length is salt dependent. The reaction was set up as described in Section 2, in the presence in the presence of 50 mM KCl (right panel) or 150 mM KCl (left panel). Radioactively labeled oligonucleotide was used to monitor the process. Each reaction mixture was loaded into two lanes and separated in neutral agarose gel. One lane was dried and analyzed, while the other was excised from the gel, soaked for 60 min in denaturing buffer (50 mM NaOH, 1 mM EDTA), and run in a second dimension under denaturing conditions, then the products were examined by phosphorimaging analysis. Labeled lambda digested with *Bst*EI was used as marker (only a subset of bands is depicted in the figure).

To probe the kinetics of D-loop conversion, and the length of the products formed, we performed time-course experiments using radioactively labeled oligonucleotide D1 as a substrate in the D-loop assay. Aliquots were withdrawn from the standard reaction mixture after 1, 2.5, 5 and 10 min and quantities of D-loop and extension products were analyzed. At the start of the extension, the reaction mixture contained over 35% of D-loop substrate. After 1 min around 10% of the D-loops were extended. The elongated products were gradually formed with up to 50% of the D-loops being extended after 10 min (Fig. 3A and B), indicating that they were rapidly and efficiently extended by Pol δ.

To estimate the length of the extension products we again used radioactively labeled oligonucleotide D1 and resolved the reaction mixtures by 2D gel electrophoresis. Fig. 3C (left panel) shows a radiogram of products of a reaction with a physiological salt concentration (150 mM KCl). Under these conditions most of the extended primer (74%) migrated as a population of molecules with estimated lengths ∼200 nt (>100 nt extension of the 90-mer primer), and a small proportion had apparent lengths reaching ca. 700 nt. When the reaction was performed in the presence of 50 mM KCl, most (68%) of the products were extended to >1000 nt (Fig. 3C, right panel). Thus, extension product lengths varied from 200 nt to 1000 nt, depending on the salt concentration.

### 3.4 Comparison of Rad51-mediated D-loop extension by Pol δ and Pol η

**Fig. 4:**
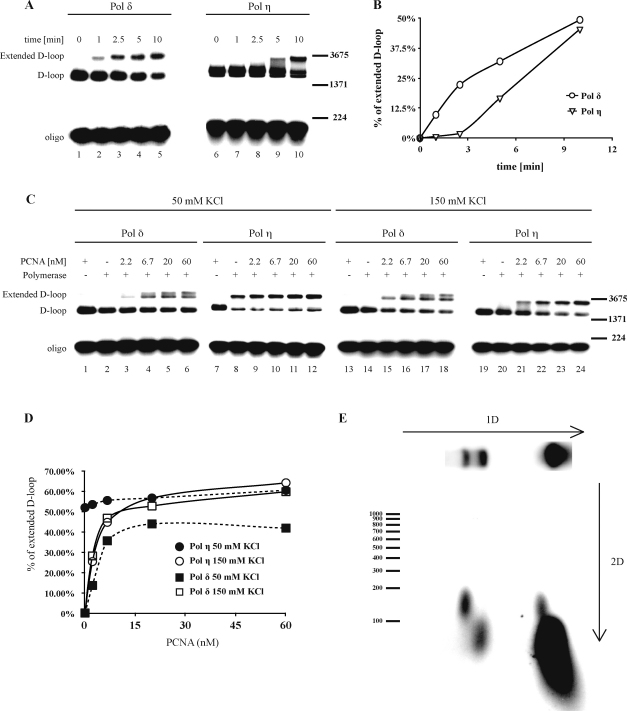
D-loop extension by Pol η and Pol δ differs due to lower processivity of the former. (A) The two polymerases exhibit different time-dependent kinetics. A time-course experiment was performed with radioactively labeled oligonucleotide to monitor the extension, in which samples were withdrawn 0, 1, 2.5, 5 and 10 min after start of the reaction. Lanes 1–5 and 6–10 represent results of experiments with Pol δ and Pol η (15 nM in both cases), respectively. (B) Quantification of the reaction described in (A). (C) Polymerases differ in PCNA requirements under low salt concentrations. Results of reactions with serial concentrations of PCNA (2.22, 6.66, 20 and 60 nM) in the presence of Pol δ (15 nM, lanes 1–6 and 13–18) or Pol η (15 nM, lanes 7–12 and 19–24) and either 50 mM KCl (lanes 1–12) or 150 mM KCl (lanes 13–24). (D) Quantification of the reaction described in (C). (E) The length of Pol η extensions was determined by 2D electrophoresis, in the presence of 150 mM KCl, using radioactively labeled oligonucleotide to monitor the extension. Labeled lambda digested with *Bst*EI was used as marker (only a subset of bands is depicted in the figure).

Next, we compared the basic properties of two polymerases associated with DNA synthesis repair, Pol δ and Pol η. For this purpose, we expressed and purified both polymerases to near-homogeneity and analyzed their ability to extend the D-loop substrate in a time-course experiment (Fig. 4A and B). In reaction mixtures containing Pol δ (lanes 1–5) products appeared within 1 min, while in mixtures with Pol η (lanes 6–10) no detectable products formed within 5 min, indicating that Pol δ is more efficient (Fig. 4B). Interestingly, however, after 10 min Pol η yielded a similar proportion of extended products (>45%) to Pol δ (Fig. 4B). The activity of the two polymerases was further compared in the presence of serial concentrations of PCNA and at two salt concentrations (50 mM and 150 mM KCl) (Fig. 4C). At the low salt concentration (50 mM KCl), Pol δ (lanes 1–6) was able to extend the primer in a PCNA concentration-dependent manner. Pol η was able to extend the primer more efficiently than Pol δ, but its activity was PCNA independent (Fig. 4C, lanes 7–12). At the high salt concentration (150 mM KCl, lanes 13–24), both polymerases showed similar PCNA-dependent D-loop extension efficiency (Fig. 4C and D). The only observed difference was that Pol δ (but not Pol η) yielded an additional, slowly migrating band representing longer extension products (Fig. 4C, lanes 4–6 and 16–18). Using 2D gel analysis to determine the length of products extended by Pol η in the D-loop extension reaction, we found that, in contrast to Pol δ, products extended by Pol η reached on average 150 nt in length in the presence of both low (50 mM) (data not shown) and high (150 mM) salt concentrations (Fig. 4E).

### 3.5 Unwinding of the extension products by Mph1 and Srs2

**Fig. 5:**
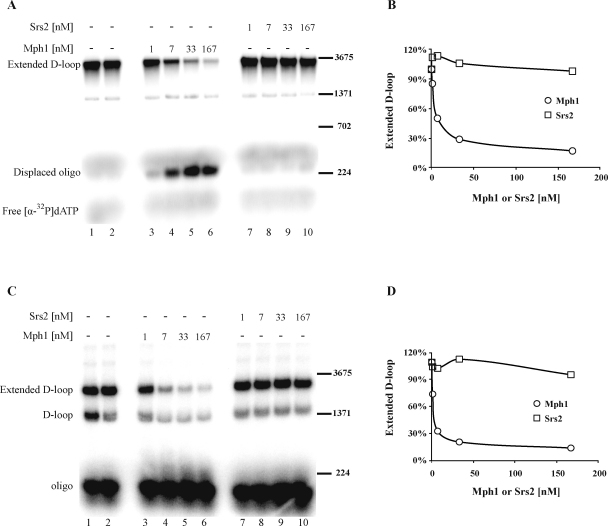
Mph1 preferably dissociates extended products. (A) A reaction mixture containing extended D-loop was incubated for 5 min at 30 °C in the presence of Mph1 (1, 7, 33 and 167 nM, lanes 3–6) or Srs2 (1, 7, 33 and 167 nM, lanes 7–10). D-loop extension was monitored by measuring the incorporation of α-[^32^P]dATP. As controls, reactions were stopped at the point when either Mph1 or Srs2 was added (lane 1) or after an additional 5 min incubation (lane 2). (B) Quantification of the reaction described in (A). (C) Results of the reaction described in (A), except that D-loop extension was monitored using radioactively labeled oligonucleotide. (D) Quantification of the reaction described in (C). Labeled lambda digested with *Bst*EI was used as marker (only a subset of bands is depicted in the figure).

The SDSA sub-pathway of DSB repair involves displacement of the newly extended strand from the D-loop followed by annealing with its original complementary strand. The actions of Srs2, Mph1 and Sgs1 helicases are implicated in SDSA [24–27], therefore we expressed and purified these helicases and tested their ability to unwind newly extended D-loops. In a first experiment (Fig. 5A) we assembled the D-loop and monitored products using [α^32^P]-dATP during 10 min extension. Serial concentrations (1, 7, 33 or 167 nM) of either Mph1 (lanes 3–6) or Srs2 (lanes 7–10) were then added and the resulting mixtures were incubated for an additional 5 min at 30 °C. The addition of 1 nM Mph1 resulted in the displacement of 15% of the extended primer. At the highest Mph1 concentration tested (167 nM) almost 90% of the extended primers were displaced (Fig. 5, lanes 3–6). In contrast, Srs2 was not able to displace the primers even at 167 nM, if anything it resulted in slight stimulation of D-loop extension (Fig. 5A and B). Similar extensions and kinetics of strand displacement were observed when the reaction was monitored using radioactively labeled oligonucleotide D1 (Fig. 5C and D). To confirm that the difference between the helicases is not due to the inability of Srs2 to unwind DNA substrates, their helicase activities were compared using a DNA substrate with a 3′ overhang. Incubation of Srs2 with such a DNA substrate resulted in the normal generation of unwound product (Supplementary Fig. 5). Furthermore, to exclude the possibility that interactions with other proteins present could inhibit unwinding of the extended primer by Srs2, we used a truncated fragment (Srs2^1–860^), which lacks the domain required for interactions with Rad51 and PCNA. Similarly to wild-type Srs2, this fragment was not capable of unwinding the extended primer (Supplementary Fig. 6, compare lanes 7–10 to lanes 11–14). Finally, Sgs1, another helicase implicated in promoting SDSA, did not have any effect on strand displacement (Supplementary Fig. 7). Taken together, these findings suggest that Mph1, but none of Srs2 or Sgs1, is fully capable of dissociating the extended primer.

## 4 Discussion

Homologous recombination is one of the major pathways for the repair of DSBs. During this process the broken DNA is sealed with a copy of an undamaged homologous sequence. The D-loop is one of the first intermediates of HR, and here we aimed to reconstitute DNA repair synthesis and strand displacement machinery *in vitro*, utilizing this substrate, to elucidate downstream events in HR. In our system, polymerase δ, PCNA and RFC were found to be absolutely required for the extension of the primer from the D-loop substrate (Fig. 1), in good agreement with previous biochemical and genetic studies [21,35,36]. The extension reaction seems to be also highly specific as *E. coli* Klenow fragment of DNA polymerase I is able to extend the D-loop only at very high concentrations (Supplementary Fig. 1B). In addition, the specificity of the reaction is supported by the ratio of substrate to protein (1:5), estimated based on the concentration of D1 oligo (8 nM) with average 35% efficiency of D-loop formation and concentration of Pol δ (15 nM), is very similar to DNA replication assay [37] and much lower to previously reported D-loop extension [21]. In comparison to the efficiency of Klenow fragment the extension by Pol δ thus should result from physiological number of polymerization cycles. Furthermore, most of the extension products were approximately 0.2 kb long, much shorter than conversion tracts observed *in vivo* [38–40], probably due to topological constraints of DNA synthesis in the plasmid system.

In addition to corroborating previously published findings, our results show that the length of extension products strongly depends on the salt concentration, as increasing it to physiological concentrations resulted in a 5-fold reduction in the size of the products (Fig. 3C). This could be due to a higher dissociation rate of the Pol δ/PCNA complex or inhibition of its ability to bind DNA, which can be overcome by using PCNA in excess of Pol δ (Fig. 4C, other data not shown). Increasing the concentration of the single-strand binding protein RPA also improved the efficiency of DNA extension, probably due to stabilization of the displaced ssDNA during the extension reaction. In addition, the data using standard model system containing singly primed ΦX-174 circular ssDNA (Supplementary Fig. 3) indicates that D-loop structure and its progression *per se* does not hinder the activities of replication proteins and also confirm the requirement of PCNA for Pol δ activity at every salt concentration tested. Alternatively, under *in vivo* conditions, additional factors might be required to overcome topological constraints or other processivity obstacles for efficient DNA extension under physiological conditions.

It has been suggested that the replicative Pol δ plays a major role in DNA repair synthesis, and it is considered to be the main polymerase associated with DNA repair extension [23,40]. *In vitro* as well as *in vivo* data indicate that a translesion polymerase, Pol η, is also capable of mediating D-loop extensions [41,42], however, further genetic studies are needed to verify the role of Pol η in repair synthesis. Here we provide a detailed comparison of the activities of these two polymerases. While Pol δ extended the primer to more than 1 kb, Pol η produced (as expected) short extension products (Fig. 4E), reflecting the differences in the biochemical properties of these polymerases, including their processivity or strand-displacement activity (Supplementary Fig. 4 [30,43,44]). DNA extension mediated by Pol η was also observed by Li et al. [21], however, our data indicate that at the reported salt concentration the DNA extension is PCNA-independent (Fig. 4C), in accordance with previous analyses of human Pol η activity using synthetic D-loop substrate [41]. The extension is only fully dependent on PCNA at physiological salt concentration, suggesting that higher salt levels could effect processivity by destabilizing the association of Pol η with DNA template. Interestingly, despite differences in the length of the extension products and rate of product formation, we observed very similar repair extension efficiencies for both polymerases, indicating that Pol η probably mediates multiple rounds of primer extension.

After successful primer extension step of HR, repair can proceed by either the SDSA or DSBR sub-pathways. The SDSA pathway is characterized by displacement of the extended primer from the D-loop, supposedly due to helicase action. *In vivo* studies have identified several helicases (including Sgs1, Mph1 and Srs2) that reduce the generation of crossover products and promote SDSA [24,27,45,46]. Sgs1 suppresses crossovers by dissolving the double Holliday junctions formed via second-end capture into non-crossovers [47–49], but is not capable of dissociating extended D-loops in our system. However, Srs2 might promote the SDSA pathway by unwinding the invading strand from the D-loop [10,11]. While Srs2 is able to unwind synthetic D-loop structures [26 and our unpublished data], we observed no effects of Srs2 on the unwinding of Rad51-mediated D-loops [24] or on products of D-loop extension (Fig. 5). In fact, we observed a slight stabilization of extension products at low Srs2 concentrations (Fig. 5). Thus, the role of Srs2 in promoting SDSA appears to be removal of Rad51 protein from the second end of the DSB, thus preventing formation of a double Holliday Junction intermediate. Alternatively, it could influence D-loop extension by interacting with Pol32 and sumoylated PCNA [15,50–53]. Post-translational modification may also be an important factor as phosphorylation and sumoylation of Srs2 influence SDSA promotion [25]. Our data clearly demonstrate that Mph1 is fully capable of dissociating the extended strand after DNA repair synthesis, in accordance with its observed ability to unwind invading strands from D-loops [24]. Mechanisms that regulate Mph1 activity during meiosis, or whenever exchange of genetic information occurs, remain to be determined. It is possible that Mph1 transcription is down-regulated, as seen during sporulation and after alpha factor arrest, processes that precede meiosis in which transcription of Mph1 decreases 2-fold [54,55]. Post-translational modifications and/or sub-cellular re-localization might also be important regulatory processes. Other factors, such as MutSα, that have been shown to participate in the Mph1-dependent promotion of SDSA, may also modulate Mph1 activity [56]. Taken together, these findings suggest that Mph1 displaces the extended primer, whereas Srs2 and Sgs1 promote SDSA by other mechanisms.

In summary, we have described an in vitro system that offers the opportunity to unravel the molecular mechanisms and regulation of HR downstream of D-loop formation. While it has been shown that the invasion step occurs with almost equal efficiency and kinetics to those observed in gene conversion (GC), break-induced replication (BIR) and single-strand annealing (SSA) [46,56], new DNA synthesis is regulated differently in these processes. It will, therefore, be enlightening to study further the differences between these processes, and thus elucidate their specific regulatory elements, including the roles of post-translational modifications and protein–protein interactions.

## Conflict of interest statement

The authors declare that there are no conflicts of interest.

## Figures and Tables

**Table 1:** Oligonucleotides used in this study.

Name	Sequence
D1	5′AAATCAATCTAAAGTATATATGAGTAAACTTGGTCTGACAGTTACCAATGCTTAATCAGTGAGGCACCTATCTCAGCGATCTGTCTATTT3′
49N	FITC 5′AGCTACCATGCCTGCACGAATTAAGCAATTCGTAATCATGGTCATAGCT3′
22mer	5′AATTCGTGCAGGCATGGTAGCT3′
27mer	FITC 5′AGCTATGACCATGATTACGAATTGCTT3′
