# Supplementary material for: Reconstitution of DNA repair synthesis in vitro and the role of polymerase and helicase activities
Source: DNA Repair (Amst). 2011 Jun 10;10(6-2):567–76. doi: 10.1016/j.dnarep.2011.03.003 (PMC3119790; doi:10.1016/j.dnarep.2011.03.003)
Supplement: Supplementary file 1 [file mmc1.pdf]

SUPPLEMENTARY METHODS

D-loop and primer extension assays

Essentially, the D-loop assay was performed as described in Krejci *et al.* [1]. Briefly, fluorescently-labeled, radioactively-labeled or unlabeled 90-mers (3 µM nucleotides) were incubated for 5 min at 37 °C with Rad51 (1 µM) in 10 µl of buffer R (35 mM Tris-Cl pH 7.4, 2 mM ATP, 2.5 mM $MgCl_2$, 50 mM KCl, 1 mM DTT and an ATP-regenerating system consisting of 20 mM creatine phosphate and 20 µg/ml creatine kinase) then 1 µl of Rad54 (150 nM) was added and the mixture was incubated for a further 3 min at 23 °C. The reaction was initiated by adding pBluescript replicative form I (50 µM base pairs) in 1.5 µl, and the mixture was incubated for 5 min at 23 °C.

Next, RPA (660 nM), PCNA (6.66 nM), RFC (10 nM) and Pol δ (3, 15, 75 nM) were added in buffer O (20 mM Tris-Cl pH 7.5, 5 mM DTT, 0.1 mM EDTA, 150 mM KCl, 40 µg/ml BSA, 8 mM $MgCl_2$, 5 % (v/v) glycerol, 0.5 mM ATP and 75 µM each of dGTP and dCTP) and the mixture was incubated for 5 min at 30 °C. DNA synthesis was initiated by adding start buffer (75 µM dTTP and either unlabeled dATP at 75µM or 0.375 µCi [α-^{32}P]dATP in buffer O) to a 30 µl final reaction volume. After 10 min at 30 °C, reactions were stopped with SDS (0.5% final) and proteinase K (0.5 mg/ml) at 37 °C for 3 min, and loaded onto an agarose gel (0.8 % w/v). After electrophoresis the gel was dried on DE81 paper and either exposed to a phosphorimager screen, or directly scanned for fluorescent DNA with a Fuji FLA 9000 imager, followed by analysis with Multi Gauge software (Fuji).

Reactions containing Klenow fragment of *E.coli* DNA polymerase I (20, 100, 500 nM) (NEB) were performed similarly to those with Pol δ, except that RPA, RFC and PCNA were omitted.

ΦX−*based extension assay*

The assay was performed essentially as described in Langston and O'Dannell [2]. The reaction (20 μl final volume) was assembled on singly primed ΦX174 virion ssDNA (5 nM) in buffer O (20 mM Tris-Cl pH 7.5, 5 mM DTT, 0.1 mM EDTA, 70 mM KCl, 0.5 mM ATP, 40 μg/ml BSA, 8 mM $MgCl_2$, 5% glycerol and 60μM each of dGTP and dCTP), in the presence of RPA (1 μM), PCNA (10 nM), RFC (17.5 nM), Pol δ (5 nM) followed by 5 min incubation at 30°C to allow loading of PCNA on the substrate. DNA synthesis was initiated by adding start buffer (60 μM dTTP and 0,375 μCi [α-^{32}P]dATP in buffer O). After indicated time at 30°C the reactions were stopped with SDS (0.5% final) and Proteinase K (0.5 mg/mL), and loaded onto agarose gel (0.8% (w/v)). After electrophoresis the gel was dried on DE81 paper, exposed to phosphorimager screen, scanned in Fuji FLA 9000 imager and analyzed with the Multi Gauge software (Fuji).

Oligo-based extension assay

The fluorescently-labeled 5' overhang (prepared from oligos 49N and 27-mer, Table 1) (100 nM) was incubated with either Pol δ or Pol η, (0.3, 0.9, 2.7, and 8.1 nM) for 3 min at 30 ºC in buffer B (25 mM Tris-Cl pH 7.5, 0.2 mg/ml BSA, 1 mM DTT, 60 mM KCl, 8 mM $MgCl_2$, 100 μM dNTPs). The reaction was stopped with SDS (0.5% final) and Proteinase K (0.5 mg/mL), and incubated at 37 ºC for 5 min. Before loading onto a 20 % PAGE denaturing gel, samples were boiled for 5 min in an equal volume of loading buffer (90 % (v/v) formamide, 0.002 % (w/v) Orange C). After electrophoresis, fluorescent DNA species were visualized in a Fuji FLA 9000 imager with Multi Gauge software (Fuji).

DNA helicase assay

The assay was performed essentially as described by Van Komen *et al.* [3]. Briefly, Srs2, Srs2$^{1\text{-}860}$ (5, 10, 20 nM) or Mph1 (0.5, 1, 2 nM) were incubated with the 3' overhang (prepared from oligos

49N and 22-mer, Table 1, 10 nM), for 5 min at 30 ºC in 10 µl of buffer H (30 mM Tris-HCl, pH 7.6, 2.5 mM $MgCl_2$, 2 mM ATP, 100 mM KCl, 1 mM dithiothreitol, and 100 µg/ml bovine serum albumin) containing an ATP-regenerating system consisting of 20 mM creatine phosphate and 20 µg/ml creatine kinase. The reaction was stopped with SDS (0.5% final) and Proteinase K (0.5 mg/mL), and DNA species were separated on a 10 % (w/v) native PAGE gel in 1xTBE buffer. Fluorescent DNA species were visualized in a Fuji FLA 9000 imager and analyzed with Multi Gauge software (Fuji).

SUPPLEMENTARY REFERENCES

[1] L. Krejci, S. Van Komen, Y. Li, J. Villemain, M.S. Reddy, H. Klein, T. Ellenberger, P. Sung, DNA helicase Srs2 disrupts the Rad51 presynaptic filament, Nature 423 (2003) 305-309.

[2] Langston, L.D. and O'Donnell, M. (2006) DNA polymerase delta is highly processive with proliferating cell nuclear antigen and undergoes collision release upon completing DNA. J Biol Chem, **283**, 29522-29531.

[3] Van Komen, S., Reddy, M.S., Krejci, L., Klein, H. and Sung, P. (2003) ATPase and DNA helicase activities of the *Saccharomyces cerevisiae* anti-recombinase Srs2. J Biol Chem, **278**, 44331-44337.

SUPPLEMENTARY FIGURE LEGENDS

Supplementary Figure 1. A) Titration of factors required for DNA repair synthesis. The reaction was set up as described. Different concentrations of PCNA (0.66, 6.6, 66 and 666 nM; lanes 5-8), RPA (0.66, 6.6, 66 and 666 nM, lanes 9-12) and Pol δ (0.16, 1.6, 16 and 160 nM; lanes 13-16) were used in individual reactions. Control experiments indicate proteins that were omitted from the reaction (lanes 1-4). RFC complex was present at a constant concentration of 10 nM. After deproteinization, the samples were separated on an agarose gel, dried and subjected to

phosphorimaging analysis. **B**) Pol δ extends D-loop more efficiently than Klenow fragment. The reaction was set up as described in supplementary methods. Increasing concentration of Pol δ (3, 15, 75 nM; lanes 3-5) and Klenow fragment (20, 100, 500 nM; lanes 6-8) was tested. Lane 1 and 2 represents control reaction where Pol δ and PCNA were omitted, respectively. Position of DNA size marker is indicated.

Supplementary Figure 2. Order of addition of RPA plays role only during D-loop formation. Experiments were set up as described in Material and methods (section 2.5). RPA (666 nM) was added together with Rad51 (lane 2), Rad54 (lane 3), dsDNA (lane 4), before (lane 5) and after (lane 6) PCNA loading, as indicated in the figure. Lane 1 represents a reaction where RPA was omitted. Position of DNA size marker is indicated.

Supplementary Figure 3. Loaded PCNA is crucial for DNA synthesis in ΦX-based system. **A**) PCNA needs to be loaded to stimulate DNA synthesis on singly primed ΦX DNA. An order of addition experiment was performed where Pol δ (20 nM) was included together (lane 3-5), after (lane 6-8), or before (lane 9-11) addition of PCNA (6.66 nM)/RFC (10 nM). Samples were withdrawn at 1, 2.5 and 5 min, respectively. In control reactions, indicated factors were omitted (lane 1 and 2). **B**) PCNA was required at every salt concentration. A titration of salt (40, 100, 120, 150 and 190 mM KCl) was performed in the absence of PCNA (lane 1-5) or in the presence of PCNA (lanes 6-10). Position of DNA size marker is indicated.

Supplementary Figure 4. Comparison of Pol η and Pol δ processivities. A primer annealed to a 5' overhang template DNA (0.1 μM) was incubated with increasing amounts (0.3, 0.9, 2.7 and 8.1 nM) of Pol δ (lanes 2-5) or Pol η (lanes 6-9) for 3 min at 30°C. Extended fluorescently-labeled primer

was separated on denaturing 20 % PAGE gel and analyzed. Lane 1 represents a control with no protein.

Supplementary Figure 5. Comparison of Mph1 and Srs2 helicase activities. The 3' overhang DNA substrate (10 nM) was incubated in the presence of Mph1 (0.5, 1 and 2 nM; lanes 2-4), Srs2 (5, 10 and 20 nM; lanes 5-7) or $Srs2^{1\text{-}860}$ (5, 10 and 20 nM; lanes 8-10) for 5 min at 30 ºC in the presence of ATP. Lane 1 represents a control with no protein. The % of unwound DNA is indicated at the bottom of the gel.

Supplementary Figure 6. The interaction with Rad51 and/or PCNA does not affect the inability of Srs2 to unwind extended D-loop products. The reaction containing extended D-loop was incubated for 5 min at 30 ºC in the presence of Mph1 (1, 7, 33 and 167 nM; lanes 3-6), Srs2 (1, 7, 33 and 167 nM; lanes 7-10) or $Srs2^{1\text{-}860}$ (1, 7, 33 and 167 nM; lanes 11-14). D-loop extension was monitored by incorporation of α-[^{32}P]dATP. As a control, reactions were stopped either at the point when Mph1 or Srs2s was added (lane 1) or after an additional 5 min incubation (lane 2). Position of DNA size marker is indicated.

Supplementary Figure 7. Sgs1 does not dissociate the extended products. A reaction mixture containing extended D-loop was incubated for 5 min at 30 ºC in the presence of Mph1 (1, 7, 33 and 167 nM; lanes 2-5) or Sgs1 (1, 7, 33 and 167 nM; lanes 7-10). D-loop extension was monitored by measuring the incorporation of α-[^{32}P]dATP. Control reactions were stopped at the point when either Mph1 or Sgs1 was added (lanes 1 and 6). Position of DNA size marker is indicated.

Supplementary figure 1

A

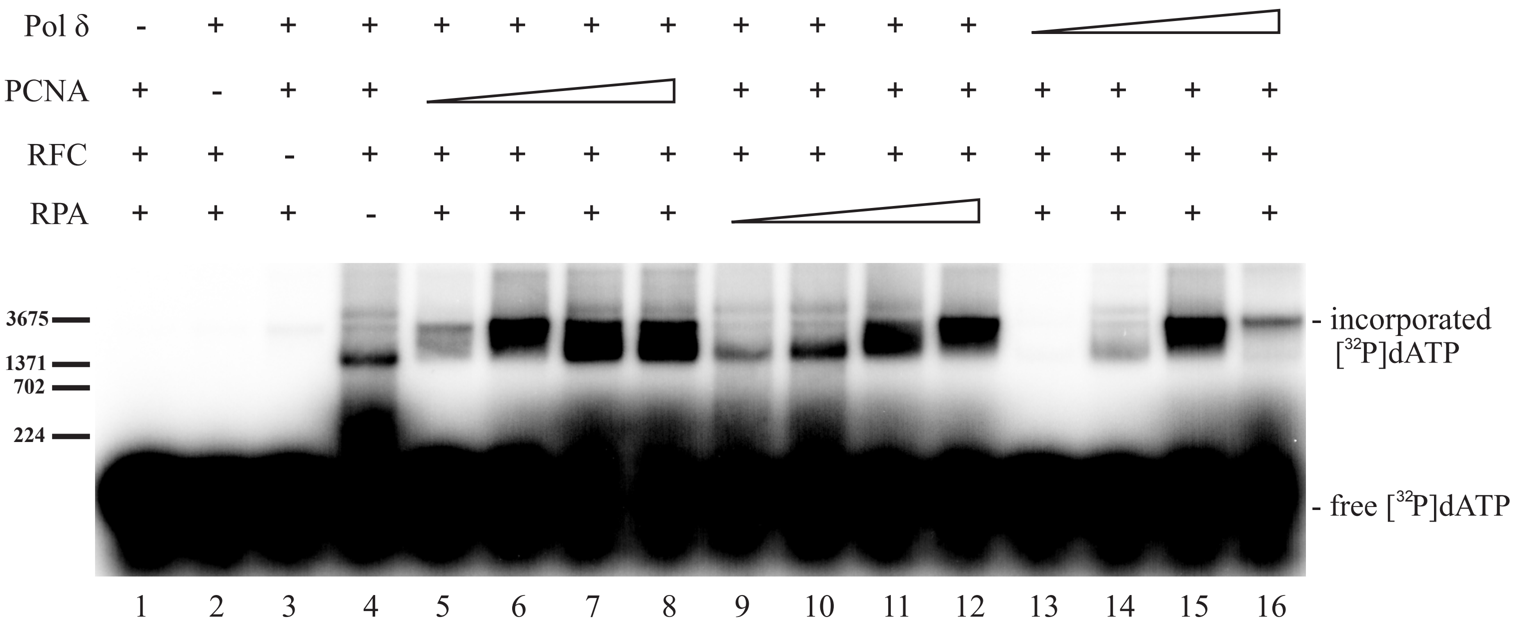

B

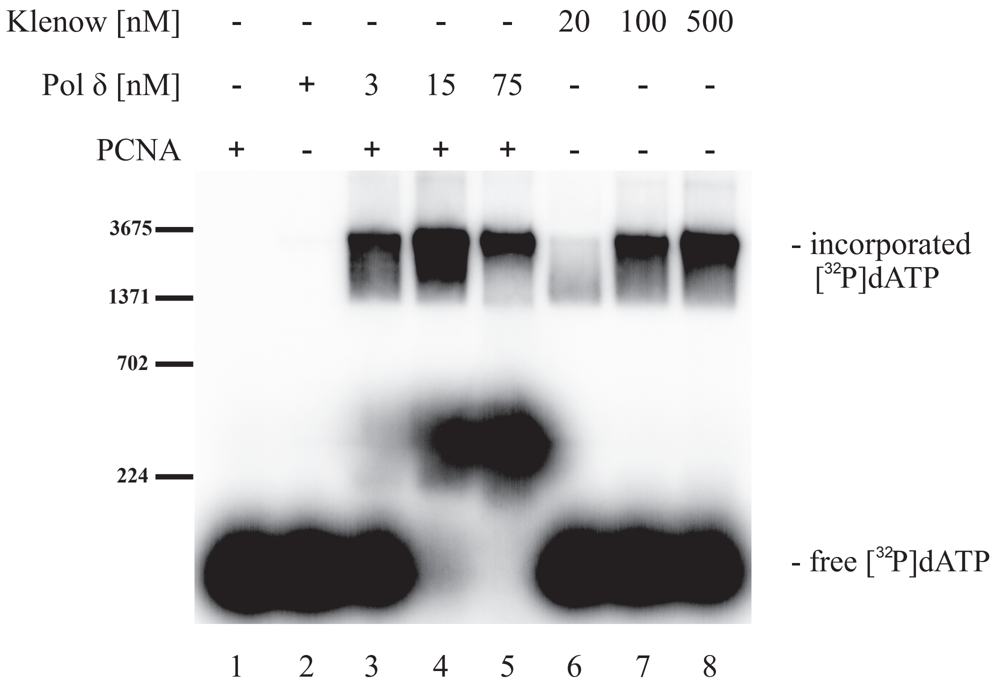

Supplementary fugure 2

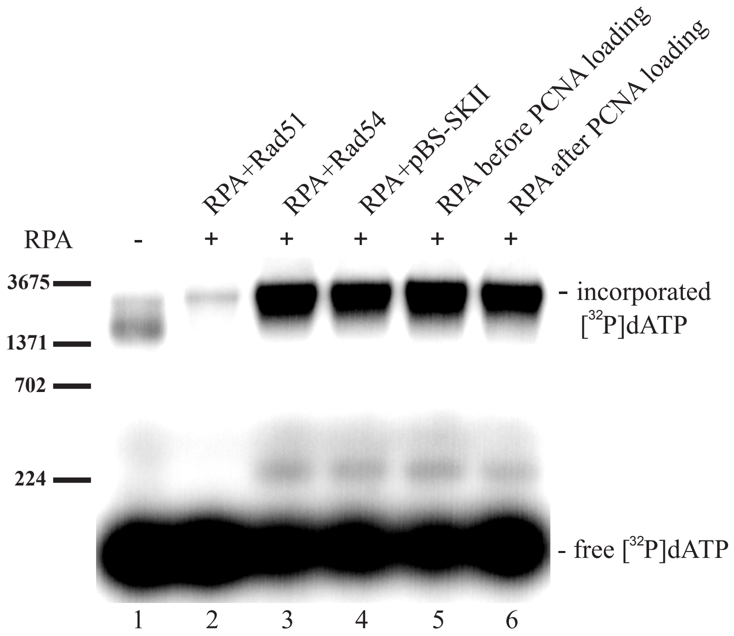

A

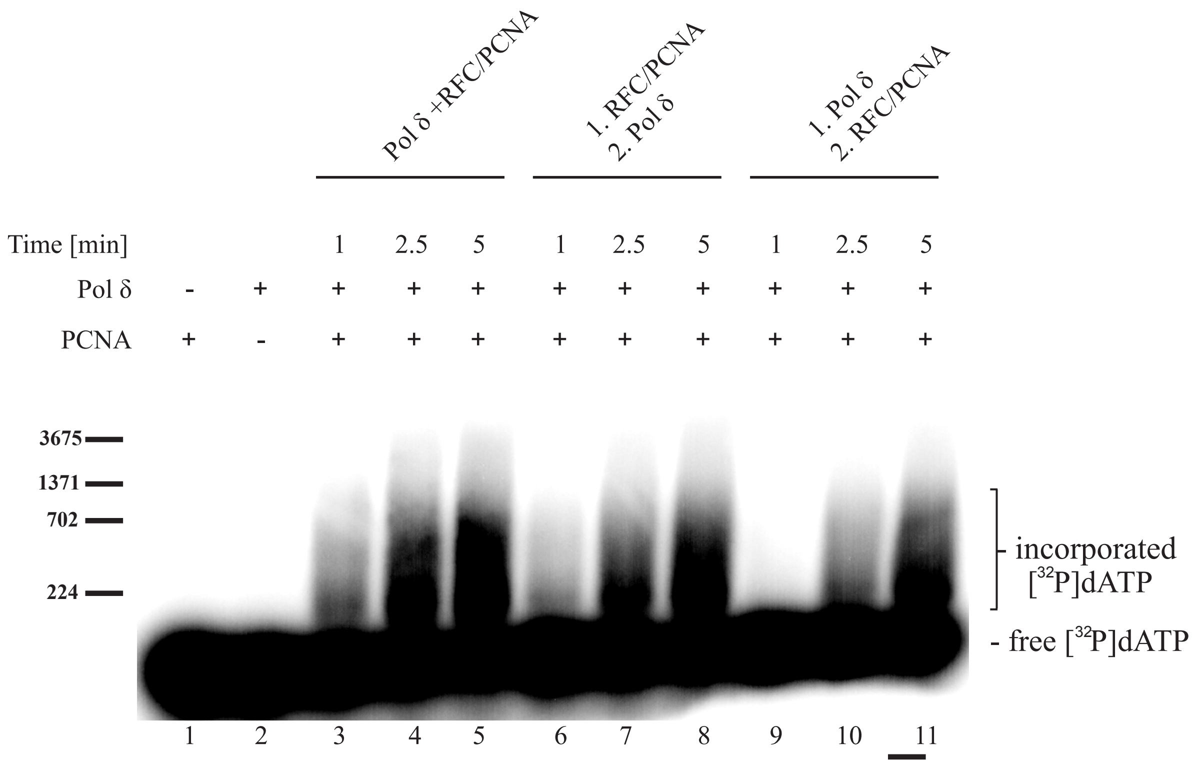
Pol δ +RFC/PCNA
1. RFC/PCNA
2. Pol δ
1. Pol δ
2. RFC/PCNA
Time [min] 1 2.5 5 1 2.5 5 1 2.5 5
Pol δ - + + + + + + + + + +
PCNA + - + + + + + + + + +
3675
1371
702
224
incorporated [32P]dATP
- free [32P]dATP
1 2 3 4 5 6 7 8 9 10 11

B

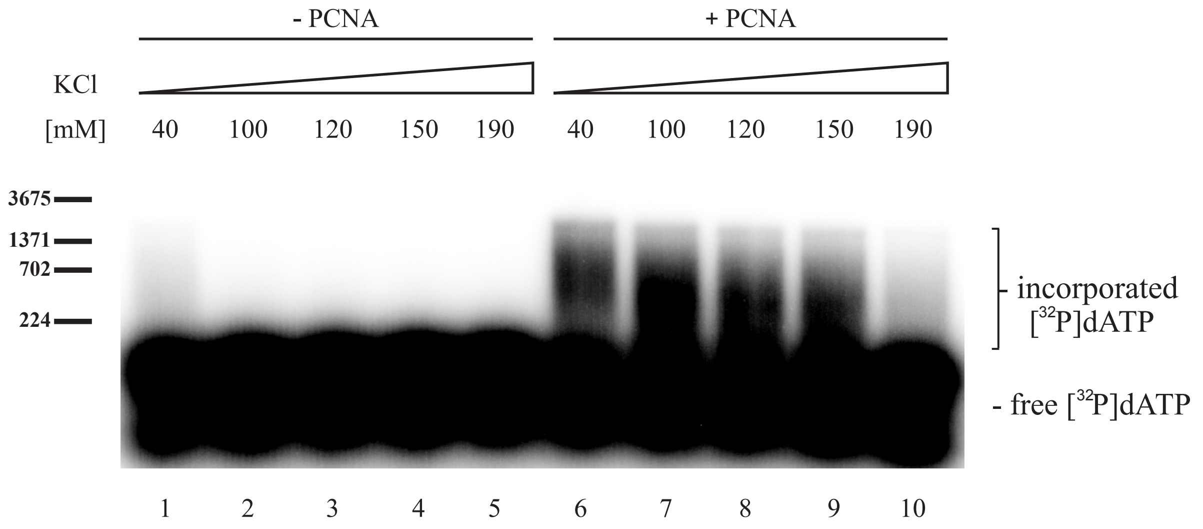
- PCNA
+ PCNA
KCl
[mM] 40 100 120 150 190 40 100 120 150 190
3675
1371
702
224
incorporated [32P]dATP
- free [32P]dATP
1 2 3 4 5 6 7 8 9 10

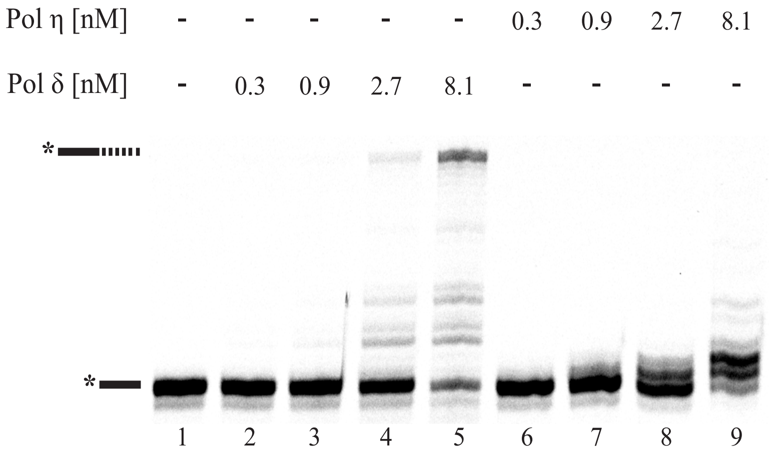
Pol η [nM] - - - - - 0.3 0.9 2.7 8.1
Pol δ [nM] - 0.3 0.9 2.7 8.1 - - - -
*
*
1 2 3 4 5 6 7 8 9

Supplementary figure 5

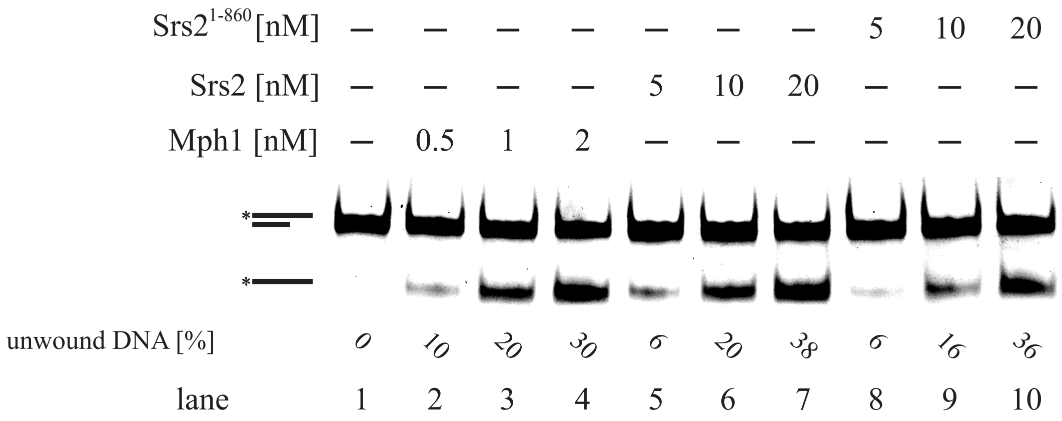

Supplementary figure 6

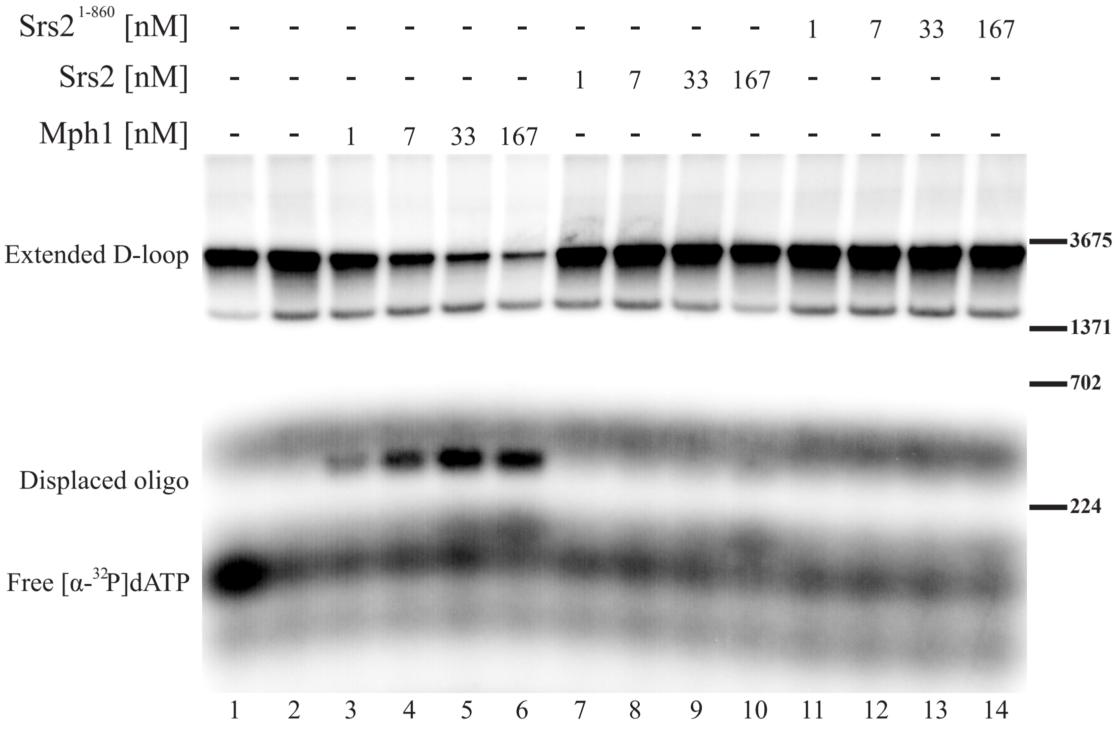

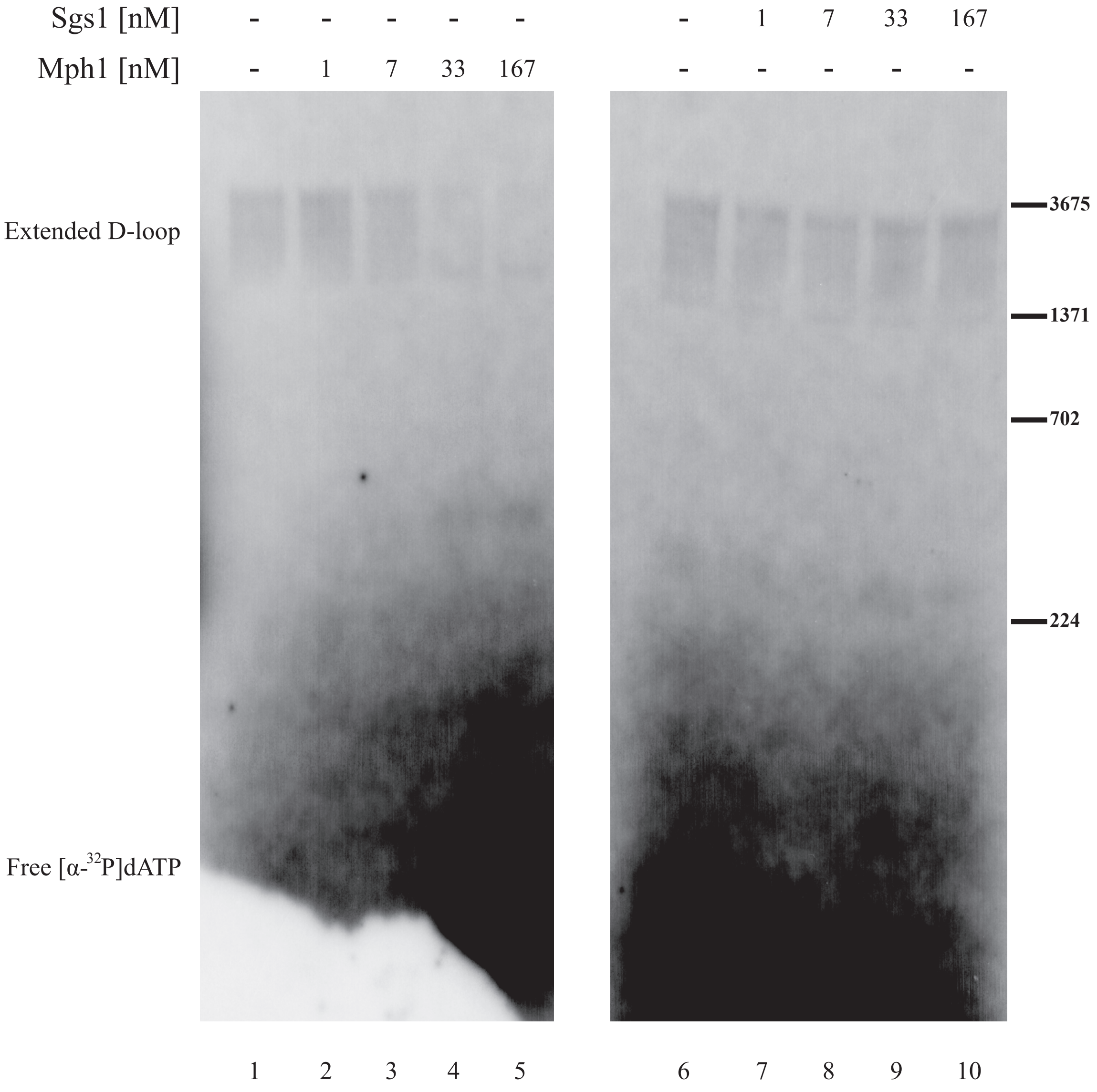

Sgs1 [nM] - - - - - - 1 7 33 167
Mph1 [nM] - 1 7 33 167 - - - - -
Extended D-loop
Free [α-32P]dATP
3675
1371
702
224
1 2 3 4 5 6 7 8 9 10
